# TCC: an R package for comparing tag count data with robust normalization strategies

**DOI:** 10.1186/1471-2105-14-219

**Published:** 2013-07-09

**Authors:** Jianqiang Sun, Tomoaki Nishiyama, Kentaro Shimizu, Koji Kadota

**Affiliations:** 1 Graduate School of Agricultural and Life Sciences, The University of Tokyo, 1-1-1 Yayoi, Bunkyo-ku, Tokyo 113-8657, Japan; 2 Advanced Science Research Center, Kanazawa University, 13-1 Takara-machi, Kanazawa 920-0934, Japan

## Abstract

**Background:**

Differential expression analysis based on “next-generation” sequencing technologies is a fundamental means of studying RNA expression. We recently developed a multi-step normalization method (called TbT) for two-group RNA-seq data with replicates and demonstrated that the statistical methods available in four R packages (*edgeR*, *DESeq*, *baySeq*, and *NBPSeq*) together with TbT can produce a well-ranked gene list in which true differentially expressed genes (DEGs) are top-ranked and non-DEGs are bottom ranked. However, the advantages of the current TbT method come at the cost of a huge computation time. Moreover, the R packages did not have normalization methods based on such a multi-step strategy.

**Results:**

*TCC* (an acronym for Tag Count Comparison) is an R package that provides a series of functions for differential expression analysis of tag count data. The package incorporates multi-step normalization methods, whose strategy is to remove potential DEGs before performing the data normalization. The normalization function based on this DEG elimination strategy (DEGES) includes (i) the original TbT method based on DEGES for two-group data with or without replicates, (ii) much faster methods for two-group data with or without replicates, and (iii) methods for multi-group comparison. *TCC* provides a simple unified interface to perform such analyses with combinations of functions provided by *edgeR*, *DESeq*, and *baySeq*. Additionally, a function for generating simulation data under various conditions and alternative DEGES procedures consisting of functions in the existing packages are provided. Bioinformatics scientists can use *TCC* to evaluate their methods, and biologists familiar with other R packages can easily learn what is done in *TCC*.

**Conclusion:**

DEGES in *TCC* is essential for accurate normalization of tag count data, especially when up- and down-regulated DEGs in one of the samples are extremely biased in their number. *TCC* is useful for analyzing tag count data in various scenarios ranging from unbiased to extremely biased differential expression. *TCC* is available at http://www.iu.a.u-tokyo.ac.jp/~kadota/TCC/ and will appear in Bioconductor (http://bioconductor.org/) from ver. 2.13.

## Background

High-throughput sequencing (HTS), also known as next-generation sequencing (NGS), is widely used to identify biological features such as RNA transcript expression and histone modification to be quantified as tag count data by RNA sequencing (RNA-seq) and chromatin immunoprecipitation sequencing (ChIP-seq) analyses [1,2]. In particular, differential expression analysis based on tag count data has become a fundamental task for identifying differentially expressed genes or transcripts (DEGs). Such count-based technology covers a wide range of gene expression level [3-6]. Several R [7] packages have been developed for this purpose [8-14].

In general, the procedure for identifying DEGs from tag count data consists of two steps: data normalization and identification of DEGs (or gene ranking), and each R package has its own methods for these steps. For example, the R package *edgeR*[8] uses a global scaling method, the trimmed mean of M values (TMM) method [15], in the data normalization step and an exact test for the negative binomial (NB) distribution [16] in the identification step. The estimated normalization factors are used within the statistical model for differential analysis and gene lists ranked in ascending order of *p*-value (or the derivative) are produced. Naturally, a good normalization method combined with a DEG identification method, should produce well-ranked gene lists in which true DEGs are top ranked and non-DEGs are bottom ranked according to the confidence or degree of differential expression (DE). Recent studies have demonstrated that the normalization method has more impact than the DEG identification method on the gene list ranking [17,18].

Note that the normalization strategies employed by most R packages assume that there is an approximately balanced proportion of DEGs between the compared samples (i.e., unbiased DE) [19]. However, a loss of function of histone modification enzymes will lead to a biased distribution of DEGs between compared conditions in the corresponding ChIP-seq analysis; i.e., there will be data with biased DE. As a result, methods assuming unbiased DE will not work well on data with biased DE. To normalize data that potentially has various scenarios (including unbiased and biased DE), we recently proposed a multi-step normalization procedure called TbT [17]. TbT consists of three steps: data normalization using TMM [15] (step 1), DEG identification by using an empirical Bayesian method implemented in the *baySeq* package [9] (step 2), and data normalization using TMM [15] after eliminating the estimated DEGs (step 3) comprising the TMM-*baySeq*-TMM pipeline. Different from conventional methods, our multi-step normalization strategy can eliminate the negative effect of potential DEGs before the normalization in step 3.

While the three-step TbT normalization method performed well on simulated and real two-group tag count data with replicates [17], it is practically possible to make different choices for the methods in each step. A more comprehensive study regarding better choices for the DEG elimination strategy (DEGES) is needed. To our knowledge, only the R package, *TCC* (from Tag Count Comparison), provides tools to perform multi-step normalization methods based on DEGES. Our work presented here enables differential expression analysis of tag count data without having to worry much about biased distributions of DEGs.

## Implementation

**Figure 1:**
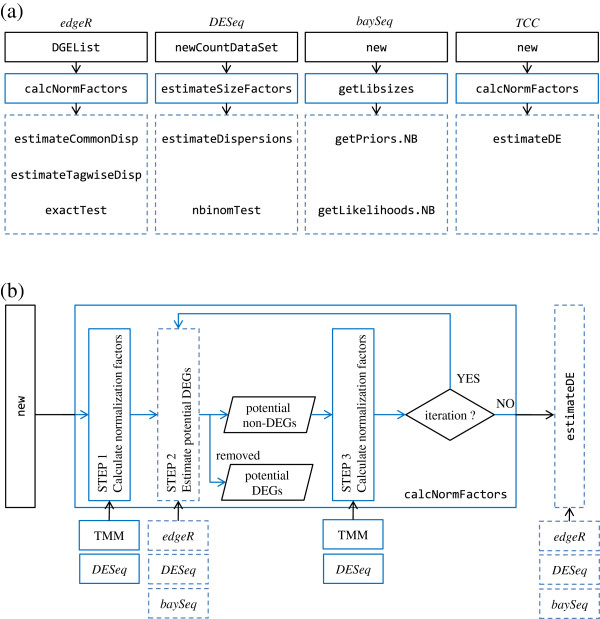
**DEGES-based analysis pipelines in *TCC. *(a)** Main functions for obtaining DE results from tag count data in individual packages (*edgeR*, *DESeq*, *baySeq*, and *TCC*). The analysis pipelines of the packages can be roughly divided into two steps after importing the input data (black squares): (i) calculating normalization factors (blue solid squares) and (ii) estimating degrees of DE for each gene (blue dashed squares). **(b)** Outline of the DEGES-based normalization methods implemented in *TCC*. The key concept of DEGES in our calcNormFactors function is to remove data flagged as potential DEGs in step 2 before calculating normalization factors in step 3. Note that steps 2 and 3 in DEGES can be repeatedly performed in order to obtain more robust normalization factors and the function accepts many iterations *n* (i.e., *n* = 0 ~ 100).

The *TCC* package was developed in the R statistical environment. This is because R is widely used and the main functionalities in *TCC* consist of combinations of functions from the existing R/Bioconductor [20] packages (i.e., *edgeR*[8], *DESeq*[10], and *baySeq*[9]). Since the main purpose (identification of DEGs from tag count data) of these three packages is essentially the same as that of *TCC* and many users may be experienced in their use, we will illustrate the main functionalities of *TCC* by contrasting them with the corresponding functions in those packages (see Figure 1). While *TCC* employs Object Oriented Programming design utilizing the R5 reference class, it has interface functions that do not change the object passed as the argument in order to be compatible with the semantics of the standard R environment. Detailed documentation for this package is provided as a vignette:

$$\mathrm{vignette}\left(”\mathrm{TCC}”\right)$$

### Preparations

Differential expression analysis between compared samples based on tag count data typically starts by preparing two objects: i) a count table matrix where each row indicates a gene, each column a sample, and each cell the number of counts (or reads) mapped to each gene in each sample and ii) a vector that indicates which group each sample belongs to. These data are stored in a *TCC* class object using the new function. Similar functions of other packages are the DGEList function in the *edgeR* package, the newCountDataSet function in the *DESeq* package, the new function in the *baySeq* package, and so on (see Figure 1a). Consider, for example, a matrix object hypoData consisting of 1,000 rows and six columns and a numeric vector group consisting of six elements, i.e., (1, 1, 1, 2, 2, 2). The first three samples in the matrix are from Group 1 (G1), and the others are from Group 2 (G2). The *TCC* class object is constructed as follows:

$$\begin{array}{l} \mathrm{library}\left(\mathrm{TCC}\right)\\ \mathrm{data}\left(\mathrm{hypoData}\right)\\ \mathrm{group}<-c\left(1,1,1,2,2,2\right)\\ \mathrm{tcc}\;<-\mathrm{new}\left(”\mathrm{TCC}”,\mathrm{hypoData},\mathrm{group}\right) \end{array}$$

### Normalization

#### Normalization of two-group count data with replicates

When obtaining normalization factors from count data with replicates, users can select a total of six combinations (two normalization methods × three DEG identification methods) coupled with an virtually arbitrary number of iterations (*n* = 0, 1, 2, …, 100) in our DEGES-based normalization pipeline (Figure 1b). Here, we describe two representative choices (DEGES/TbT and iDEGES/*edgeR*).

##### DEGES/TbT

The *TCC* package provides robust normalization methods based on the DEGES recently proposed by Kadota et al. [17]. The original three-step normalization method (TbT) is performed by specifying the two major arguments (norm.method and test.method) as follows:

$$\begin{array}{l} \;\\ \begin{array}{c} \mathrm{tcc}<-\mathrm{calcNormFactors}(\mathrm{tcc},\mathrm{norm}.\mathrm{method}=”\mathrm{tmm}”,\\ \;\mathrm{test}.\mathrm{method}=”\mathrm{bayseq}”,\\ \;\mathrm{iteration}\;=\;1) \end{array} \end{array}$$

In relation to the other DEGES-based methods, we will call the method “DEGES/TbT” for convenience. As mentioned in ref. [17], the multi-step normalization can be repeated until the calculated normalization factors converge. The iterative version of the DEGES/TbT (iDEGES/TbT) can be described as a TMM-(*baySeq*-TMM)_*n*_ pipeline with *n* > = 2. Accordingly, the TMM normalization method [15] and the DEGES/TbT can be described as pipelines with *n* = 0 and 1, respectively, and *n* can be specified by the iteration argument.

##### DEGES/edgeR

A major disadvantage of the TbT method is the long time it requires to calculate the normalization factors. This requirement is due to the empirical Bayesian method implemented in the *baySeq* package. To alleviate this problem, a choice of alternative methods should be provided for step 2. For instance, using the exact test [16] in *edgeR* in step 2 enables the DEGES normalization pipeline to be much faster and entirely composed of functions provided by the *edgeR* package. The three-step DEGES normalization pipeline (we will refer to this as the TMM-(*edgeR*-TMM)_*n*_ pipeline with *n* = 1 or DEGES/*edgeR*, for convenience) can be performed by changing the test.method argument to “edger”.

The TbT pipeline automatically calculates the percentage of DEGs (*P*_DEG_) by virtue of its use of *baySeq*. In contrast, a reasonable threshold for defining potential DEGs should also be provided when using the exact test in *edgeR* (or the NB test in *DESeq*). Here, we define the threshold as an arbitrary false discovery rate (FDR) with a floor value for *P*_DEG_. The default is FDR < 0.1 (i.e., FDR = 0.1), and the default floor *P*_DEG_ value is 5% (i.e., floorPDEG = 0.05); different choices are possible. For example, in the case of the default settings, *x*% (*x* > 5%) of the top-ranked potential DEGs are eliminated in step 2 if the percentage (= *x*%) of genes satisfying FDR < 0.1 is over 5%.

Although iDEGES/TbT would not be practical because of its long computation time, the iterative version of DEGES*/edgeR* (iDEGES/*edgeR* with *n* > = 2) is potentially superior to both non-iterative DEGES*/edgeR* and DEGES/TbT. A suggested choice of *n* = 3, consisting of seven steps, can be performed by changing the iteration argument (i.e., iteration = 3) as follows:

$$\begin{array}{ll} \mathrm{tcc}<\;-\mathrm{calcNormFactors} & \left(\mathrm{tcc},\mathrm{norm}.\mathrm{method}=”\mathrm{tmm}”,\right)\\ & \mathrm{test}.\mathrm{method}=”\mathrm{edger}”,\\ & \mathrm{FDR}=0.1,\mathrm{floorPDEG}=0.05,\\ & \mathrm{iteration}\;=\;\left(3\right)\; \end{array}$$

The suggested number of iterations is determined from the results of iDEGES/TbT; it is a number at which the accuracies of DEG identifications corresponding to the calculated normalization factors converge [17]. The number of iterations for iDEGES/*edgeR* is determined in the same way (see the Results and discussion section).

#### Normalization of two-group count data without replicates

Most R packages are designed primarily for analyzing data including biological replications because the biological variability has to be accurately estimated to avoid spurious DE calls [21]. In fact, the functions for the DEG identification method implemented in *edgeR* (i.e., the exact test; ver. 3.0.4) do not allow one to perform an analysis without replicates, even though the TMM normalization method in the package can be used regardless of whether the data has replicates or not. Although the *edgeR* manual provides users with some ideas on how to perform the DE analysis, it is practically difficult to customize the analysis with DEGES to data without replicates.

However, there are still cases in which we have to perform DE analysis of tag count data without replicates. When obtaining normalization factors from two-group count data without replicates, users can select from a total of four combinations (two normalization methods × two DEG identification methods) and a virtually arbitrary number of iterations (*n* = 0, 1, 2, …, 100) in our DEGES-based normalization pipeline. That is, the calcNormFactors function with the norm.method = “deseq” or “tmm” and test.method = “deseq” or “bayseq” can be selected. A pipeline (iDEGES/*DESeq*; the *DESeq*-(*DESeq*-*DESeq*)_*n*_ pipeline with *n* = 3) using functions in the *DESeq* package for analyzing two-group count data without replicates can be performed by changing the two arguments (norm.method and test.method) as follows:

$$\begin{array}{ll} \mathrm{tcc}<-\mathrm{calcNormFactors} & \left(\mathrm{tcc},\mathrm{norm}.\mathrm{method}=”\mathrm{deseq}”,\right)\\ & \mathrm{test}.\mathrm{method}=”\mathrm{deseq}”,\\ & \mathrm{FDR}=0.1,\mathrm{floorPDEG}=0.05,\\ & \left(\mathrm{iteration}\;=\;3\right) \end{array}$$

#### Normalization of multi-group count data with replicates

Many R packages (including *edgeR*, *DESeq*, and *baySeq*) support DE analysis for multi-group tag count data. *TCC* provides DEGES-based normalization methods for such data by virtue of the three packages that are internally used in *TCC*. Similar to the analysis of two-group count data with replicates, users can select from a total of six combinations (two normalization methods × three DEG identification methods) and a virtually arbitrary number of iterations (*n* = 0, 1, 2, …, 100) when obtaining normalization factors from multi-group data with replicates.

#### Retrieving normalized data

The calculated normalization factors can be obtained from tcc$norm.factors. Similar functions are the calcNormFactors function in the *edgeR* package and the estimateSizeFactors function in the *DESeq* package, (Figure 1a). Note that the terminology used in *DESeq* (i.e., size factors) is different from that used in *edgeR* (i.e., effective library sizes) and ours. The effective library size in *edgeR* is calculated as the library size multiplied by the normalization factor. The size factors in the *DESeq* package are comparable to the *normalized* effective library sizes, wherein the summary statistics for the effective library sizes are adjusted to one. Our normalization factors, which can be obtained from tcc$norm.factors, have the same names as those in *edgeR*. Since biologists are often interested in such information [19], we provide the getNormalizedData function for retrieving the normalized data. The normalized data can directly be used as, for example, the input data of another package *NOISeq*[13].

### Differential expression

The goal of the analysis would be to obtain a list of DEGs. To this end, we provide the estimateDE function. The function internally calls the corresponding functions implemented in three packages: exactTest in *edgeR*, nbinomTest in *DESeq*, and getLikelihoods.NB in *baySeq* (Figure 1). If the user wants to perform the DE method implemented in *edgeR* and to determine the genes having an FDR threshold of 10% as DEGs, one can do as follows:

$$\mathrm{tcc}<-\mathrm{estimateDE}\left(\mathrm{tcc},\mathrm{test}.\mathrm{method}=”\mathrm{edger}”,\mathrm{FDR}=0.1\right)$$

A similar analysis based on DE methods in *DESeq* or *baySeq* can be performed by changing the test.method parameter to “deseq” or “bayseq”. The results of the DE analysis are stored in the *TCC* class object. The summary statistics for the top-ranked genes can be retrieved by using the getResult function. In general, the identified DEGs at FDR < 0.1 should be up-regulated in either G1 or G2. The plot function generates an M-A plot, where “M” indicates the log-ratio (i.e., M = log_2_G2 - log_2_G1) and “A” indicates the log average read count (i.e., A = (log_2_G2 + log_2_G1)/2) based on the normalized count data.

### Generation of simulation data

As demonstrated in our previous study [17], the DEGES-based normalization methods implemented in *TCC* theoretically outperform the other normalization methods when the numbers of DEGs between groups are biased. However, the validation of the biased DE in real data is very difficult in practice [19]. Thus, the performance of methods handling biased DE needs to be evaluated using simulation data. The simulateReadCounts function generates simulation data with various conditions. Currently, this function can generate simulation data analyzed in the TbT paper [17], thereby enabling further comparisons of our DEGES-based methods with methods developed by other researchers in the near future. For example, the hypoData object, a hypothetical count dataset in *TCC*, was generated by using this function with the following arguments:

$$\begin{array}{ll} \mathrm{tcc}<-\mathrm{simulateReadCounts} & \left(\mathrm{Ngene}=1000,\mathrm{PDEG}=0.2,\right)\\ & \mathrm{DEG}.\mathrm{assign}\;=\;c\left(0.9,0.1\right),\;\\ & \mathrm{DEG}.\mathrm{foldchange}\;=\;c\left(4,4\right),\;\\ & \left(\mathrm{replicates}\;=\;c\left(3,3\right)\right) \end{array}$$

The simulation conditions for comparing two groups (G1 vs. G2) with biological replicates are as follows: (i) the number of genes is 1,000 (i.e., Ngene = 1000), (ii) the first 20% of genes are DEGs (PDEG = 0.2), (iii) the first 90% of the DEGs are up-regulated in G1 and the remaining 10% are up-regulated in G2 (DEG.assign = c(0.9, 0.1)), (iv) the levels of DE are four-fold in both groups (DEG.foldchange = c(4, 4)), and (v) there are a total of six samples (three biological replicates for G1 and three biological replicates for G2) (replicates = c(3, 3)). The empirical distribution of read counts is built from *Arabidopsis* data in *NBPSeq*[12].

The output of the simulateReadCounts function is stored in the *TCC* class object with information about the simulation conditions and is therefore ready-to-analyze. This function can generate several kinds of simulation data, such as those for comparing four groups (Groups 1–4) with replicates and those for comparing two groups without replicates. See the vignette for details.

## Results and discussion

Accurate data normalization is essential for obtaining well-ranked gene lists from tag count data. Similar to other R packages such as *edgeR*, the *TCC* package has functionalities for DE analysis of tag count data. Of these functionalities, *TCC* provides multi-step normalization methods (including DEGES/TbT [17]) that internally use the functions implemented in *edgeR*, *DESeq*, and *baySeq*. Here, we demonstrate that the DEGES-based normalization methods are more effective than the methods implemented in the other packages. All analyses were performed using R (ver. 2.15.2) and Bioconductor [20]. Execution times were measured on a Linux system (CentOS release 6.2 (Final), Intel® Xeon® E5-4617 (2.9 GHz) 24 CPU, and 512 GB memory). The versions of major R libraries were *TCC* ver. 1.1.99, *edgeR* ver. 3.0.4, *DESeq* ver. 1.10.1, and *baySeq* ver. 1.12.0.

Following our previous study [17], we here demonstrate the performance of these methods by using the same evaluation metric, simulation framework, and real experimental datasets. We use the area under the receiver operating characteristic (ROC) curve (i.e., AUC) as a means of comparison. The simulation conditions are as follows: 5-25% of the genes are DEGs (*P*_DEG_ = 5–25%), 50–90% of the DEGs are up-regulated in G1 compared to G2 (*P*_G1_ = 50–90%), and the levels of DE are four-fold in both groups. The count dataset consists of 70,619 unique small RNAs (sRNAs) and a total of four *Arabidopsis thaliana* leaf samples (i.e., two wild-type [WT1 and WT2] and two RNA-dependent RNA polymerase 6 (RDR6) knockout [KO1 and KO2] samples) [9]. This dataset was originally analyzed with *baySeq*. The data has 657 provisional true DE sRNAs (i.e., *P*_DEG_ = 657 / 70,619 = 0.93%), and all of the sRNAs can be regarded as up-regulated in the wild-type (i.e., *P*_G1_ = 100%). This is because they uniquely match tasRNA, which is produced by RDR6, and they are down-regulated in RDR6 mutants. In addition to the RDR6 knockout dataset, we also analyze four other experimental datasets (called “*sultan*” [4], “*gilad*” [22], “*maqc*” [23], and “*katz.mouse*” [24]) obtained from the ReCount database [25]. The four datasets are used for evaluating the DEGES-based methods aimed at two-group count data with replicates.

### Simulation data with replicates (sensitivity, specificity, and computation time)

We assessed the performance of a total of six normalization methods: (a) TMM, (b) DEGES/TbT, (c) DEGES/*edgeR*, (d) iDEGES/*edgeR*, (e) iDEGES/TDT (i.e., the TMM-(*DESeq*-TMM)_*n*_ pipeline), and (f) iDEGES/*DESeq*. The ranked gene lists were obtained using the individual DEG identification methods in three packages (*edgeR*, *DESeq*, and *baySeq*), together with normalization factors calculated from each normalization method. Accordingly, a total of 18 combinations (six normalization methods × three DEG identification methods) were evaluated. Table 1 shows the average AUC values of 100 trials between the ranked gene lists and the truth for various simulation conditions (*P*_DEG_ = 5–25% and *P*_G1_ = 50–90%). While the *n* iterations for the three iDEGES-based methods roughly require an *n*-fold computation time, the improvement due to increasing the number of iterations plateaued around *n* = 3 when performing iDEGES/TbT [17]. Therefore, we decided to show AUC values for the three iDEGES-based methods with *n* = 3.

**Table 1:** Average AUC values for simulation data with replicates

**DE method**	***edgeR***	***edgeR***	***edgeR***	***DESeq***	***DESeq***	***DESeq***	***baySeq***	***baySeq***	***baySeq***
***P***_**G1**_	**50%**	**70%**	**90%**	**50%**	**70%**	**90%**	**50%**	**70%**	**90%**
(a) TMM									
*P*_DEG_ = 5%	90.30	90.30	90.18	89.09	89.08	88.92	89.43	89.42	89.22
*P*_DEG_ = 15%	90.30	90.12	89.63	89.15	88.91	88.26	89.54	89.28	88.66
*P*_DEG_ = 25%	90.40	89.89	88.26	89.27	88.62	86.82	89.63	89.07	87.25
(b) DEGES/TbT									
*P*_DEG_ = 5%	90.30	90.31	90.28	89.09	89.12	89.07	89.43	89.47	89.41
*P*_DEG_ = 15%	90.30	90.25	90.26	89.15	89.07	89.01	89.54	89.48	89.49
*P*_DEG_ = 25%	**90.41**	90.27	89.89	89.27	89.07	88.61	89.63	89.56	89.22
(c) DEGES/*edgeR*									
*P*_DEG_ = 5%	90.29	90.32	90.28	89.09	89.13	89.08	89.43	89.48	89.41
*P*_DEG_ = 15%	**90.30**	90.25	90.26	89.15	89.07	89.02	89.54	89.48	89.50
*P*_DEG_ = 25%	90.40	90.28	89.91*	89.27	89.08	88.64	89.63	89.56	89.24
(d) iDEGES/*edgeR*									
*P*_DEG_ = 5%	90.29	**90.32**	90.28	89.09	89.13	89.08	89.43	89.48	89.42
*P*_DEG_ = 15%	90.30	**90.25**	90.29*	89.15	89.08	89.06	89.54	89.49	89.54
*P*_DEG_ = 25%	90.40	90.30*	90.04*	89.27	89.11	88.80	89.63	89.60	89.42
(e) iDEGES/TDT									
*P*_DEG_ = 5%	**90.30**	90.32	**90.28**	89.09	89.12	89.09	89.43	89.48	89.42
*P*_DEG_ = 15%	90.30	90.23	90.20	89.15	89.05	88.93	89.54	89.45	89.40
*P*_DEG_ = 25%	90.40	90.25	89.82	89.27	89.04	88.53	89.63	89.52	89.13
(f) iDEGES/*DESeq*									
*P*_DEG_ = 5%	90.30	90.31	90.28	89.09	89.13	89.09	89.43	89.48	89.43
*P*_DEG_ = 15%	90.30	90.24	90.21	89.15	89.05	88.95	89.54	89.45	89.42
*P*_DEG_ = 25%	90.40	90.26	89.86	89.27	89.05	88.58	89.63	89.53	89.18

Average AUC values (%) of 100 trials for each simulation condition are shown. Simulation data contain a total of 10,000 genes: *P*_DEG_% of genes is for DEGs, *P*_G1_% of *P*_DEG_ in G1 is higher than in G2, and each group has three biological replicates (i.e., G1_rep1, G1_rep2, G1_rep3, G2_rep1, G2_rep2, and G2_rep3). A total of nine conditions (three *P*_DEG_ values × three *P*_G1_ values) are shown. The highest AUC value for each condition is in bold. AUC values with asterisks indicate significant improvements (*p*-value < 0.01, paired *t*-test) compared with DEGES/TbT. We used a bootstrap resampling size of 10,000 in *baySeq* when performing the normalization (i.e., *baySeq* at step 2 in the DEGES/TbT) and 2,000 when performing the DEG identification after normalization (i.e., *baySeq* in the *XXX*-*baySeq* combination).

DEGES/*edgeR* performed comparably to TbT, whereas iDEGES/*edgeR* outperformed the others, irrespective of the choice of DEG identification method (*edgeR*, *DESeq*, and *baySeq*) after normalization. That is, iDEGES/*edgeR* followed by any DEG identification method *YYY* (termed the iDEGES/*edgeR*-*YYY* combination) performed the best among the six normalization methods. These results demonstrate that the alternative DEGES approaches (iDEGES/*edgeR*) implemented in *TCC* generally outperform the original DEGES approach (i.e., DEGES/TbT). In other words, the use of the exact test [16] implemented in *edgeR* is sufficient to determine the potential DEGs to be eliminated. Advantageous characteristics for the exact test also revealed themselves after performing any normalization method: Comparing the three DEG identification methods (*edgeR*, *DESeq*, and *baySeq*), we see that the *XXX*-*edgeR* have the highest AUC values. Overall, iDEGES/*edgeR*-*edgeR* performed the best. It should be noted that, however, the AUC values for the pipeline look very close to those of the original recommendation (i.e., DEGES/TbT-*edgeR*) [17], e.g., 90.30% for iDEGES/*edgeR*-*edgeR* and 90.27% for DEGES/TbT-*edgeR* under one simulation condition of *P*_DEG_ = 25% and *P*_G1_ = 70%.

Although the AUC values for the individual combinations are under 0.0042% of the standard deviation (see “Sheet 1” in Additional file 1) and are statistically significant (*p* < 0.01; see Table 1), some researchers may think the current recommendation (iDEGES/*edgeR*-*edgeR*) does not look compelling. We do not argue the fact that iDEGES/*edgeR* performs comparably to DEGES/TbT (or DEGES/*edgeR*), regarding the absolute AUC values (i.e., sensitivity and specificity). We rather want to emphasize that iDEGES/*edgeR* outperforms DEGES/TbT in terms of computation time (Table 2). This table shows that iDEGES/*edgeR* takes roughly three-times longer than DEGES/*edgeR* but it is over 100 times faster than DEGES/TbT. In light of the absolute computation times (shorter than 10 seconds), iDEGES/*edgeR* finishes execution within one minute. Although the TMM normalization method has the shortest computation time, its AUC values are clearly lower than those of the others, especially when the *P*_G1_ value is displaced from 50%. An evaluation based on sensitivity and specificity should take precedence over one based on the computation time. These results show that iDEGES/*edgeR* is good for analyzing two-group tag count data with replicates because of its sensitivity, specificity, and practical computation time.

**Table 2:** Average computation times for obtaining normalization factors

***P***_**G1**_	**50%**	**70%**	**90%**
(a) TMM			
*P*_DEG_ = 5%	0.13	0.18	0.22
*P*_DEG_ = 15%	0.16	0.19	0.15
*P*_DEG_ = 25%	0.17	0.14	0.15
(b) DEGES/TbT			
*P*_DEG_ = 5%	1492.92	1423.55	1573.23
*P*_DEG_ = 15%	1556.93	1510.17	1408.46
*P*_DEG_ = 25%	1527.08	1483.09	1543.80
(c) DEGES/*edgeR*			
*P*_DEG_ = 5%	3.04	3.08	3.24
*P*_DEG_ = 15%	3.08	3.05	2.89
*P*_DEG_ = 25%	2.99	2.92	3.05
(d) iDEGES/*edgeR*			
*P*_DEG_ = 5%	8.80	8.95	9.53
*P*_DEG_ = 15%	8.94	8.91	8.39
*P*_DEG_ = 25%	8.75	8.39	8.92
(e) iDEGES/TDT			
*P*_DEG_ = 5%	17.92	17.54	18.24
*P*_DEG_ = 15%	19.85	19.45	18.74
*P*_DEG_ = 25%	21.17	20.74	21.16
(f) iDEGES/*DESeq*			
*P*_DEG_ = 5%	17.88	17.54	18.34
*P*_DEG_ = 15%	19.96	19.43	18.72
*P*_DEG_ = 25%	21.17	20.75	21.27

Average computation times (in seconds) of 100 trials for the six normalization methods in Table 1 are shown. The results of DEGES/TbT were obtained by using a suggested parameter setting for performing bootstrap resampling (i.e., samplesize = 10000).

### Simulation data with replicates (effect of iteration)

Next, let us show the effect of iterations in the iDEGES approach to see whether the iteration can truly produce a convergent result or not. Table 3 summarizes the results under three simulation conditions (*P*_G1_ = 50, 70, and 90% with a fixed *P*_DEG_ value of 20%). Of a total of 300 trials, iDEGES/*edgeR* yielded 251 convergent and 49 non-convergent (i.e., cyclic) results. We got similar results for the three other iDEGES methods (iDEGES/TbT, iDEGES/TDT, and iDEGES/*DESeq*), irrespective of the simulation conditions. These results clearly indicate that the iDEGES-based normalization methods do not always produce convergent normalization factors, contrary to previous expectations [17].

**Table 3:** Result of the iDEGES approach does not necessarily convergent

***P***_**G1**_	**50%**	**70%**	**90%**	**Total**
(a) iDEGES/TbT				
convergent	69	70	83	222
cyclic	31	30	17	78
(b) iDEGES/*edgeR*				
convergent	86	86	79	251
cyclic	14	14	21	49
(c) iDEGES/TDT				
convergent	82	81	84	247
cyclic	18	19	16	53
(d) iDEGES/*DESeq*				
convergent	99	97	100	296
cyclic	1	3	0	4

The numbers of convergent and non-convergent (cyclic) results of 100 trials under *P*_DEG_ = 20% are shown: (a) iDEGES/TbT, (b) iDEGES/*edgeR*, (c) iDEGES/TDT, and (d) iDEGES/*DESeq*. We defined the trial as ‘convergent’ if potential DEGs estimated in the (*N*_C_ + 1)^th^ iteration was the same as those in the (*N*_C_)^th^ iteration and the number of iteration *n* required for obtaining the convergent result as *N*_C_ (*N*_C_ > = 1). We defined the trial as ‘cyclic’ if potential DEGs estimated in the (*i* + *N*_P_)^th^ iteration were the same as those in the *i*^th^ iteration and the cycle as *N*_P_ (*N*_P_ > = 2).

Of practical interest when using the iDEGES approach is the number of iterations required for obtaining a convergent result. We defined *n* as *N*_C_ if the potential DEGs estimated at the (*N*_C_ + 1)^th^ iteration are the same as those in the (*N*_C_)^th^ iteration. The distribution of *N*_C_ values for the four iDEGES-based methods are given in Additional file 2. In our trials, the maximum *N*_C_ value was 8 (see “Sheet 1” in Additional file 2). The distribution suggests that the iDEGES approach with *n* = 8 could be sufficient for obtaining convergent results under various simulation scenarios. However, the improvement had by iDEGES/*edgeR* with *n* > = 4 compared with that with *n* = 3 is actually negligible despite the requirement of additional computation time (see “Sheet 3” in Additional file 2). Therefore, the use of our iDEGES pipeline with *n* = 3 can be recommended for reducing useless computation time.

We observed that the number of iterations needed for obtaining convergent results (the *Nc* value) tends to increase when the degree of biased DE is high (*P*_G1_ > 50%): the average *N*_C_ values for the iDEGES/*edgeR* under the three conditions of *P*_G1_ = 50, 70, and 90% were 2.17, 3.14, and 3.60, respectively (see “Sheet 1” in Additional file 2). This is reasonable because, in the case of the simulation with *P*_G1_ > > 50%, a relatively large number of *n* in the TMM-(*edgeR*-TMM)_*n*_ pipeline is theoretically needed for obtaining accurate normalization factors and because, in the case of the simulation with *P*_G1_ = 50%, the theoretical *P*_G1_ value obtained from the potential DEGs in any iDEGES-based pipeline at *n* = 1 is 50% (i.e., no need to apply the iDEGES approach to such data).

Now, let us briefly discuss the non-convergent results. All the non-convergent results showed cyclic characteristics, that is, there exists a same set of potential DEGs estimated in both the *i*^th^ and the (*i* + *N*_P_)^th^ iterations within a trial (*N*_P_ > = 2). We found that the most frequent *N*_P_ value was 2 (see “Sheet 1” in Additional file 2). For example, iDEGES/*edgeR* yielded 83.7% (41/49) non-convergent results with *N*_P_ = 2. This result indicates that two different sets of potential DEGs are alternately eliminated in the *i*^th^ and (*i* + 1)^th^ iterations. Note that different normalization methods do not seem to produce consistent results (convergent (C) or non-convergent (P)) within a trial. Under one simulation condition (*P*_DEG_ = 20% and *P*_G1_ = 90%), for example, iDEGES/*edgeR* and iDEGES/TbT got 21 and 17 non-convergent results, respectively. Of these, only three trials (i.e., the 8th, 10th, and 61th) showed the same non-convergent results (see “Sheet 2” in Additional file 2).

We confirmed that the *P*_DEG_ values (satisfying FDR < 0.1) originally estimated by three methods (iDEGES/*edgeR*, iDEGES/TDT, and iDEGES/*DESeq*) in every iteration in Table 3 were above the predefined floor value of 5%, indicating that the floor value of 5% for *P*_DEG_ has no effect on whether the results converge or not. The iterative method can be viewed as a discrete dynamical system since the number of state is finite and determined by the combination of genes as potential DEGs. Oscillations in the trajectory are common phenomena in such dynamical systems. Thus, a method based on an optimality criterion should be developed to select the best point in the cycle after detecting an oscillation. Nevertheless, we observed an overall improvement regarding non-convergent results when more iterations were used.

### Simulation data without replicates

*TCC* also provides DEGES-based methods for normalizing two-group data without replicates. As described previously, the DEG identification method (i.e., the exact test) in *edgeR* does not allow for an analysis without replicates. Accordingly, we evaluated a total of eight *XXX*-*YYY* combinations (*XXX* = *DESeq*, iDEGES/*DESeq*, iDEGES/TDT, and DEGES/TbT; *YYY* = *DESeq* and *baySeq*). Here, the *DESeq*-*DESeq* combination indicates the original procedure in *DESeq*. Different from the results for the data with replicates, iDEGES/*DESeq* (and iDEGES/TDT) performed better than DEGES/TbT in this case (see “Sheet 1” in Additional file 3). The same trend can be seen in the accuracies of the estimated DEGs: the accuracies calculated from *baySeq* (i.e., DEGES/TbT) were clearly inferior to those from *DESeq*, irrespective of the choice of normalization method (see “Sheet 2” in Additional file 3). The advantageous characteristics of the NB test in *DESeq* become apparent when we compare the two DEG identification methods (*DESeq* and *baySeq*) for any normalization method *XXX*: the *XXX*-*DESeq* combination outperforms the *XXX*-*baySeq* combination. These results indicate that the higher AUC values of the iDEGES/*DESeq*-*DESeq* are primarily by virtue of a well-ranked gene list produced from the NB test and that constructing a model based on bootstrap resampling employed in *baySeq* is difficult in a non-replicate situation.

We observed that the numbers of potential DEGs satisfying FDR < 0.1 in the iDEGES/*DESeq* were nearly zero (i.e., the estimated *P*_DEG_ values were 0%) in all of the simulations, although the *P*_DEG_ values were 5–25%. This is reasonable because any attempt to work without replicates will lead to conclusions of very limited reliability: *DESeq* employs a conservative approach to prevent spurious DE calls. Accordingly, a predefined floor *P*_DEG_ value (= 5%) was used; i.e., 5% of the top-ranked genes were not used when calculating the normalization factors in the iDEGES/*DESeq* and iDEGES/TDT methods. These facts indicate that (i) iDEGES/*DESeq* performs almost as well as the original normalization method in *DESeq* when the floor *P*_DEG_ value is decreased from the default (= 5%), (ii) the methods perform equally well when the floor *P*_DEG_ value of 0% for iDEGES/*DESeq* is used, and (iii) iDEGES/*DESeq* (and iDEGES/TDT) with a floor value of *x*% tends to work better when analyzing simulation data with the same *P*_DEG_ value. Note that we set the floor *P*_DEG_ value to 5% in order to obtain certain differences between the iDEGES-based methods and the original *DESeq*, but we should consider the appropriate choice of the parameter in the future. Results of iterations in the iDEGES approach (i.e., convergent or non-convergent) when analyzing two-group data without replicates were given in Additional file 4.

### Real data (*Arabidopsis* RDR6 knockout dataset)

Here, we describe the results for a real dataset with replicates, i.e., {WT1, WT2} vs. {KO1, KO2}. The experiment was originally reported in Ref. [9]. Following the evaluation scheme in Table 1, we calculated the AUC values by using the 18 combinations shown in Table 4. Consistent with the results shown in Table 1, we found that the *XXX*-*edgeR* combination (i.e., the use of the DEG identification method in *edgeR* after any normalization method) was the best, and consequently, we recommend using this combination to analyze two-group data with replicates. Although the iDEGES/*edgeR*-*edgeR* combination performed the best on simulation data with replicates (see Table 1), iDEGES/*edgeR* did not distinguish itself among the six *XXX*-*edgeR* combinations. Note also that the AUC value for iDEGES/*edgeR* with *n* = 3 (65.70%) was inferior to that for iDEGES/*edgeR* with *n* = 1 (i.e., DEGES/*edgeR*; 69.28%), suggesting that the iterative approach had a negative impact on this TMM-(*edgeR*-TMM)_*n*_ pipeline.

**Table 4:** AUC values for an RDR6 knockout dataset

**DE method**	***edgeR***	***DESeq***	***baySeq***
TMM	68.36	61.09	60.21
DEGES/TbT	66.70	61.40	60.09
DEGES/*edgeR*	69.28	61.27	60.31
iDEGES/*edgeR*	65.70	61.34	60.86
iDEGES/TDT	65.76	60.84	60.19
iDEGES/*DESeq*	64.88	60.45	59.20

The AUC values (%) for a total of 18 combinations with default setting (i.e., a floor *P*_DEG_ of 5%) are shown.

**Figure 2:**
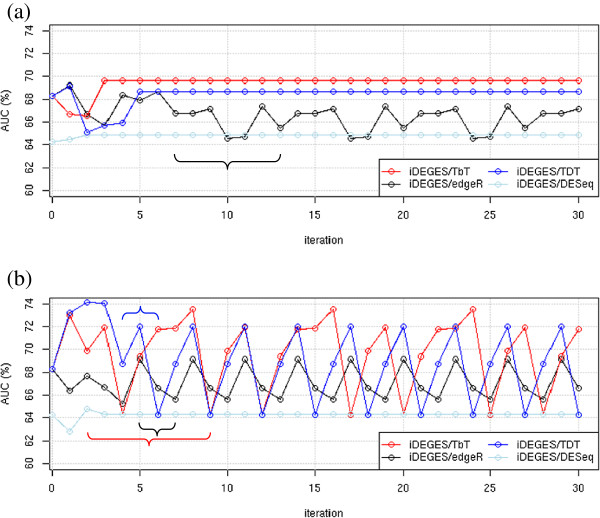
**Effect of iterations for an RDR6 knockout dataset. (a)** AUC values for four *XXX*-*edgeR* combinations with different iteration numbers (*n* = 0 ~ 30) when using a default floor *P*_DEG_ value (= 5%) are shown: *XXX* = iDEGES/TbT (red lines), iDEGES/*edgeR* (black lines) , iDEGES/TDT (blue lines) , and iDEGES/*DESeq* (light blue lines). The AUC values after convergence were 69.64% for iDEGES/TbT at the 3rd iteration and 64.88% for iDEGES/*DESeq* at the 2nd iteration. The maximum and minimum values among cycles for iDEGES/*edgeR* were 67.37% and 64.57%, respectively. Note that the AUC values for three pipelines (iDEGES/TbT, iDEGES/*edgeR*, and iDEGES/TDT) are the same (= 68.36%) when *n* = 0 because those pipelines correspond to *XXX* = TMM (i.e., the TMM-*edgeR* combination). The first cycle for the non-convergent (P) results is indicated by a brace. **(b)** AUC values when using a 10% floor *P*_DEG_ value are shown. The *N*_P_ values for iDEGES/TbT, iDEGES/*edgeR*, and iDEGES/TDT were 8, 3, and 3, respectively.

To see the effect of iteration, we investigated the changes in the AUC values for the four *XXX*-*edgeR* combinations (*XXX* = iDEGES/TbT, iDEGES/*edgeR*, iDEGES/TDT, and iDEGES/*DESeq*) when the default floor *P*_DEG_ value (= 5%) was used (Figure 2a). The figure shows that the values from the iDEGES/*edgeR* pipeline (black lines) has a cyclic characteristics with *N*_P_ = 7, and the cycle starts at the 7th iteration (i.e., *N*_S_ = 7). In contrast, the AUC values from the other pipelines had convergent characteristics: *N*c = 3 for iDEGES/TbT, 2 for iDEGES/*DESeq*, and 5 for iDEGES/TDT. Interestingly, the *P*_DEG_ values estimated by iDEGES/*edgeR*, iDEGES/TDT, and iDEGES/*DESeq* were 0% in every iteration. Because of that, the floor *P*_DEG_ value (5% default) was employed instead of the original values. Meanwhile, the estimated *P*_DEG_ values for iDEGES/TbT were above 5% in every iteration (ranging from 6.09% to 6.18%). These results indicate that the floor value of 5% for *P*_DEG_ has no effect on whether the results converge or not.

We observed that the compositions of the potential DE sRNAs were identical when the AUC values were the same (see, e.g., light blue line at *n* = 2 ~ 30). This was true even for cyclic results (e.g., the compositions obtained from iDEGES/*edgeR* at the 10th, 17th, and 24th iterations). The different AUC values, in turn, were due to the difference in the normalization factors at each iteration and the different compositions caused the next normalization factors to be different. We found that the relatively large difference in the AUC values among cycles for the non-convergent results (approximately 3% difference) were due to (i) the paucity of variations among the 657 provisional true DE sRNAs and (ii) their low expression levels. Of the 657 sRNAs, there were only 143 unique patterns of counts across the four samples (i.e., WT1, WT2, KO1, and KO2). For example, there were 91 DE sRNAs that had one tag count only in WT1, i.e., (1, 0, 0, 0) and 200 DE sRNAs that had one count only in WT2, i.e., (0, 1, 0, 0). These two count patterns occupied 44.3% of the 657 DE sRNAs. Such low-count DE sRNAs cannot be distinguished from other non-DE sRNAs if both patterns are identical. Indeed, we found that the positions for the low-count DE sRNAs in the ranked gene list varied from iteration to iteration.

Similar to the paucity of variations regarding the count vectors for the 657 DE sRNAs, we found that out of a total of 70,619 sRNAs, there were only 2,535 unique patterns of sRNA counts. This indicates that many sRNAs displayed the same degrees of DE, and therefore, their ranks (or the AUC values calculated based on the ranks) will vary if slight changes are made to the calculated normalization factors. Indeed, we observed considerably different changes in the AUC values when a higher floor *P*_DEG_ value of 10% was used (Figure 2b); e.g., the results for iDEGES/TbT converged when a 5% floor was used, but they became cyclic when 10% was used (the red lines in the figure). We also found that iDEGES/ TbT, iDEGES/*edgeR*, and iDEGES/TDT performed about the same when a 10% floor was used. These results indicate that the large difference between AUC values in this dataset might be within the error range.

All results described above were obtained with the original raw count data, in accordance with the *edgeR* and *baySeq* design. However, we should note that the current procedure is different from one recommended in the TbT paper [17], where the data was scaled to counts per million (CPM) in each sample when the DEG identification method in *baySeq* (i.e., the empirical Bayesian method) was executed. This recommendation was derived from a comparison of uncertain AUC values between the raw count data and the CPM data of this dataset, and it is now questionable. Accordingly, all the procedures implemented in the current *TCC* are based on the original raw count data.

### Real data (four ReCount datasets)

Lastly, let us show the results of four experimental datasets: three human RNA-seq datasets (called “*sultan*” [4], “*gilad*” [22], and “*maqc*” [23]) and one mouse dataset (“*katz.mouse*” [24]). These datasets were obtained from the ReCount database [25]. Different from the RDR6 dataset and simulated datasets, we do not know the true DEGs for the four datasets, indicating that we cannot calculate the AUC values. We therefore investigated the effect of iteration regarding the potential DEGs to be eliminated (i.e., convergent or non-convergent). Consistent with the RDR6 result, we observed several cyclic (P) characteristics for the ReCount datasets (see “Sheet 2” in Additional file 5).

**Figure 3:**
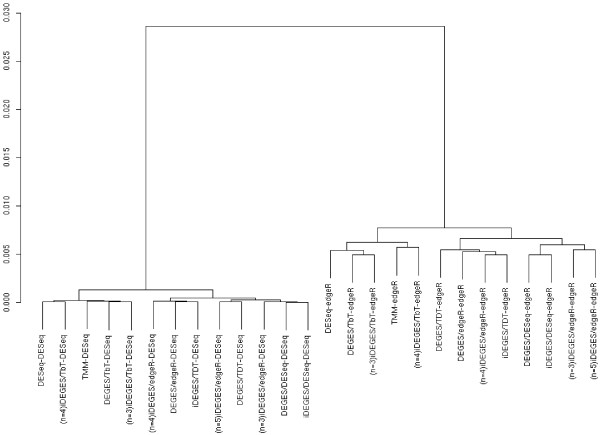
**Dendrogram of average-linkage hierarchical clustering for a *gilad *dataset.** Dendrograms for a total of 26 ranked gene lists when using a default floor *P*_DEG_ value setting (= 5%) are shown. The correlation coefficient is used as a similarity metric; the left-hand scale represents (1 - correlation coefficient). Each gene list is denoted as an “*XXX*-*YYY*” combination (the normalization method *XXX* followed by the DEG identification method *YYY*). Gene lists having iteration numbers on the left side correspond to cyclic results.

It is important to evaluate the degree of impact for the cyclic results. In the *gilad* dataset, for example, we observed cyclic results for two DEGES pipelines: iDEGES/*edgeR* (*N*_S_ = 3 and *N*_P_ = 3) and iDEGES/TbT (*N*_S_ = 3 and *N*_P_ = 2). Figure 3 shows the results of hierarchical clustering applied to a total of 26 ranked gene lists (13 *XXX*-*edgeR* and 13 *XXX*-*DESeq* combinations). Two distinct clusters can be seen: each cluster (i) consists of 13 gene lists with the same DEG identification methods after performing different normalization methods (i.e., the *XXX*-*edgeR* cluster and the *XXX*-*DESeq* cluster) and (ii) has five cyclic results (denoted as “(*iteration number*) followed by *combination name*”; e.g., “(n = 4)iDEGES/TbT-*edgeR*”). It is clear that the five cyclic results are quite similar to the eight other results. We also got similar results for another dataset (see Additional file 6). These results suggest that cyclic results are not of concern in practice.

## Conclusion

The R package *TCC* provides users with a robust and accurate framework to perform DE analysis of tag count data. *TCC* has an improved data normalization step, compared with existing packages such as *edgeR*. While the other normalization strategies assume that there is an approximately balanced proportion of DEGs between compared samples (i.e., unbiased DE), our multi-step DEGES-based normalization methods are designed to deal with various scenarios (including unbiased and biased DE situations): the internally used DEG identification method eliminates the effects of biases, if any, of potential DEGs. Our study demonstrated that the iDEGES/*edgeR*-*edgeR* combination can be recommended for analyzing two-group data with replicates (see Tables 1 and 2) and that the iDEGES/*DESeq*-*DESeq* can be recommended for analyzing two-group data without replicates (see Additional file 3). The success of these methods primarily owes to the high scalability of the normalization and DEG identification methods in the R packages used in *TCC*.

The functionality of *TCC* can be extended in many ways. First, the current study focuses on the analysis of two-group data, but some users may want to utilize the DEGES-based methods for data consisting of two or more groups. As can be seen in the vignette, some prototypes of DEGES-based pipelines for analyzing data in three or four groups have already been implemented. Evaluations such as these and further improvement are our next tasks. Second, the current approach of *TCC* is somewhat reminiscent of a microarray analysis of the count matrix for *genes*. We know that (i) one or more isoforms can be transcribed in a same gene region, (ii) those transcripts may have distinct expression levels, and (iii) it could lead to a DE result at the transcript level but a non-DE result at the gene level. To prevent this possible problem and fully utilize the resolution of RNA-seq data, advanced R packages (e.g., *BitSeq*[21] and *DEXSeq*[14]) have recently been developed. We believe that the use of those packages together with DEGES enables us to obtain a more reliable result, because the idea of DEGES can, of course, be applied to DE analysis at both gene-level and transcript-level resolutions.

Finally, the current DEGES-based normalization methods implemented in *TCC* only employ linear scaling normalization methods and statistical methods for identifying DEGs. This is because these scaling normalization methods do not change the shape of the original count distribution and the statistical methods for identifying DEGs in *edgeR*, *DESeq*, and *baySeq* assume the model. Technically speaking, we can construct many other pipelines consisting of, for example, a non-linear normalization method (e.g., quantile normalization [26]) and a DEG identification method originally developed for microarray analysis (e.g., WAD [27]). As we learned from the microarrays, there are suitable combinations of normalization methods and DEG identification methods [28]: we speculate that a non-linear normalization method would be incompatible with a statistical DE method. A critical evaluation of those pipelines will be of interest in the future and we will continue to offer up-to-date guidelines.

## Availability and requirements

**Project name:** TCC

**Project home page:***TCC* is available at http://www.iu.a.u-tokyo.ac.jp/~kadota/TCC/ and will appear in Bioconductor (http://bioconductor.org/) from ver. 2.13.

**Operating systems:** Platform independent

**Programming language:** R

**Other requirements:** requires the *edgeR*, *DESeq*, *baySeq*, and *ROC* packages

**License:** GPL-2

**Any restrictions to use by non-academics:** None

## Abbreviations

DE: Differential expression; DEG: Differentially expressed gene; DEGES: DEG elimination strategy; FDR: False discovery rate; CPM: Counts per million (normalization); sRNA: small RNA; tasRNA: *TAS* locus-derived small RNA; TMM: Trimmed mean of M values (method); TbT: the TMM-*baySeq*-TMM pipeline; TCC: Tag count comparison.

## Competing interests

The authors declare that they have no competing interests.

## Authors’ contributions

JS improved the *TCC* package, performed the analysis, and drafted the paper. TN drafted, maintained the *TCC* package, and refined the paper. KS supervised the critical discussion and refined the paper. KK drafted the R scripts, refined the paper, and conducted this project. All the authors read and approved the final manuscript.

## Acknowledgments

The authors thank Dr. TJ Hardcastle for providing the dataset used in the *baySeq* study. This study was supported by grants (KAKENHI 24500359 to KK and 22128008 to TN) from the Japanese Ministry of Education, Culture, Sports, Science and Technology (MEXT).

## Supplementary Material

Additional file 1 **Additional results for simulation data with replicates. Sheet 1**: Standard deviations of AUC values in Table 1 are shown. Legends are the same as given in Table 1. **Sheet 2**: Estimated values for *P*_DEG_ and accuracies of the potential DEGs are shown. Averages of 100 trials are shown. Values for (a) TMM do not exist because it does not estimate potential DEGs. Following the original TbT study, the *P*_DEG_ value for DEGES/TbT was directly obtained from the posterior probability output of *baySeq* (i.e., a floor value for *P*_DEG_ was set to 0%; floorPDEG = 0.00) and the accuracy was calculated on the basis of the estimated DEGs. The estimated *P*_DEG_ values for the other DEGES-based methods were calculated as relative numbers of genes satisfying FDR < 0.1 (i.e., FDR = 0.1). The accuracy was calculated on the basis of the potential DEGs if the *P*_DEG_ value was over 5% of the predefined floor *P*_DEG_ value (i.e., floorPDEG = 0.05) and, otherwise, the accuracy of the 5% of top-ranked genes was calculated as the surrogate DEGs. Click here for file

Additional file 2 **Effect of iterations for simulation data with replicates.** Details of the results in Table 3 are shown. **Sheet 1**: The *N*_C_ and *N*_P_ values are shown: (a) iDEGES/TbT, (b) iDEGES/*edgeR*, (c) iDEGES/TDT, and (d) iDEGES/*DESeq*. **Sheet 2:** Raw results (convergent (C) or non-convergent (P)) for each of the 100 trials are shown. The number to the right of the C (or P) indicates the *N*_C_ (or *N*_P_) value. **Sheet 3**: Average AUC values (%) for the iDEGES/*edgeR*-*edgeR*. AUC values with *n* = 1–6 for (a) all trials, (b) trials only having convergent results, and (c) trials only having non-convergent results are shown. Click here for file

Additional file 3 **Results for simulation data without replicates.** Results of four normalization methods (*XXX* = (a) *DESeq*, (b) iDEGES/*DESeq*, (c) iDEGES/TDT, and (d) DEGES/TbT) under each simulation condition are shown. **Sheet 1**: Average AUC values (%) of 100 trials and the standard deviations from the *XXX*-*DESeq* and the *XXX*-*baySeq* combinations are shown. Legends are the same as in Table 1. **Sheet 2**: Estimated values for *P*_DEG_ and accuracies of the potential DEGs are shown. Averages of 100 trials are shown. Values for (a) *DESeq* do not exist because it does not estimate potential DEGs. Other legends are the same as in Additional file 1. **Sheet 3**: Average computation times (in seconds) of 100 trials for the four normalization methods are shown. Click here for file

Additional file 4 **Effect of iterations for simulation data without replicates. Sheet 1**: Summary of convergent or non-convergent (cyclic) results of 100 trials for three iDEGES-based normalization methods: (a) iDEGES/*DESeq*, (b) iDEGES/TDT, and (c) iDEGES/TbT. **Sheet 2**: Raw results (convergent (C) or non-convergent (P)) for each of the 100 trials are shown. **Sheet 3**: Average AUC values (%) for the iDEGES/*DESeq*-*DESeq*. AUC values with *n* = 1–6 for (a) all trials, (b) trials only having convergent results, and (c) trials only having non-convergent results are shown. The characteristics of *N*_P_ = 2 for non-convergent results can be seen. Click here for file

Additional file 5 **Effect of iterations for real data. Sheet 1**: Basic information for the four ReCount datasets as well as the RDR6 dataset are shown. The dataset names except for the RDR6 one are the same as those provided in the ReCount database. The numbers in the “non-zero counts” column indicate the numbers of genes (or transcripts or rows) having non-zero counts for at least one of the compared groups. The numbers in the “unique patterns” column indicate the numbers of unique patterns among the genes having non-zero counts. **Sheet 2**: Results (convergent (C) or non-convergent (P)) of four iDEGES-based normalization methods when using floor *P*_DEG_ value settings of (a) 5% and (b) 10% are shown. The number to the right of the C (or P) indicates the *N*_C_ (or *N*_P_) value. The analysis were performed using non-zero count data. Note that the results of iDEGES/*edgeR* for the *maqc* dataset were not shown because the method cannot be applied to data without replicates. Click here for file

Additional file 6 **Dendrogram of average-linkage hierarchical clustering for a *katz.mouse *dataset.** Legends are basically the same as those in Figure 3. Gene lists having iteration numbers on the right side correspond to the results of the iDEGES/TbT-*YYY* combinations: the iDEGES/TbT pipeline for this dataset converged after the fifth iteration (i.e., *N*_C_ = 5; see “Sheet 2” in Additional file 5). It can be seen that the ranked gene lists obtained from the same combination with different iteration numbers (i.e., the five lists obtained from the iDEGES/TbT-*YYY* combination with *n* = 1 ~ 5) are quite similar to the lists obtained from the other *XXX*-*YYY* combinations if *YYY* is the same. Click here for file
