# Supplementary figures and images for: TCC: an R package for comparing tag count data with robust normalization strategies
Source: BMC Bioinformatics. 2013 Jul 9;14:219. doi: 10.1186/1471-2105-14-219 (PMC3716788; doi:10.1186/1471-2105-14-219)

## Slide 1
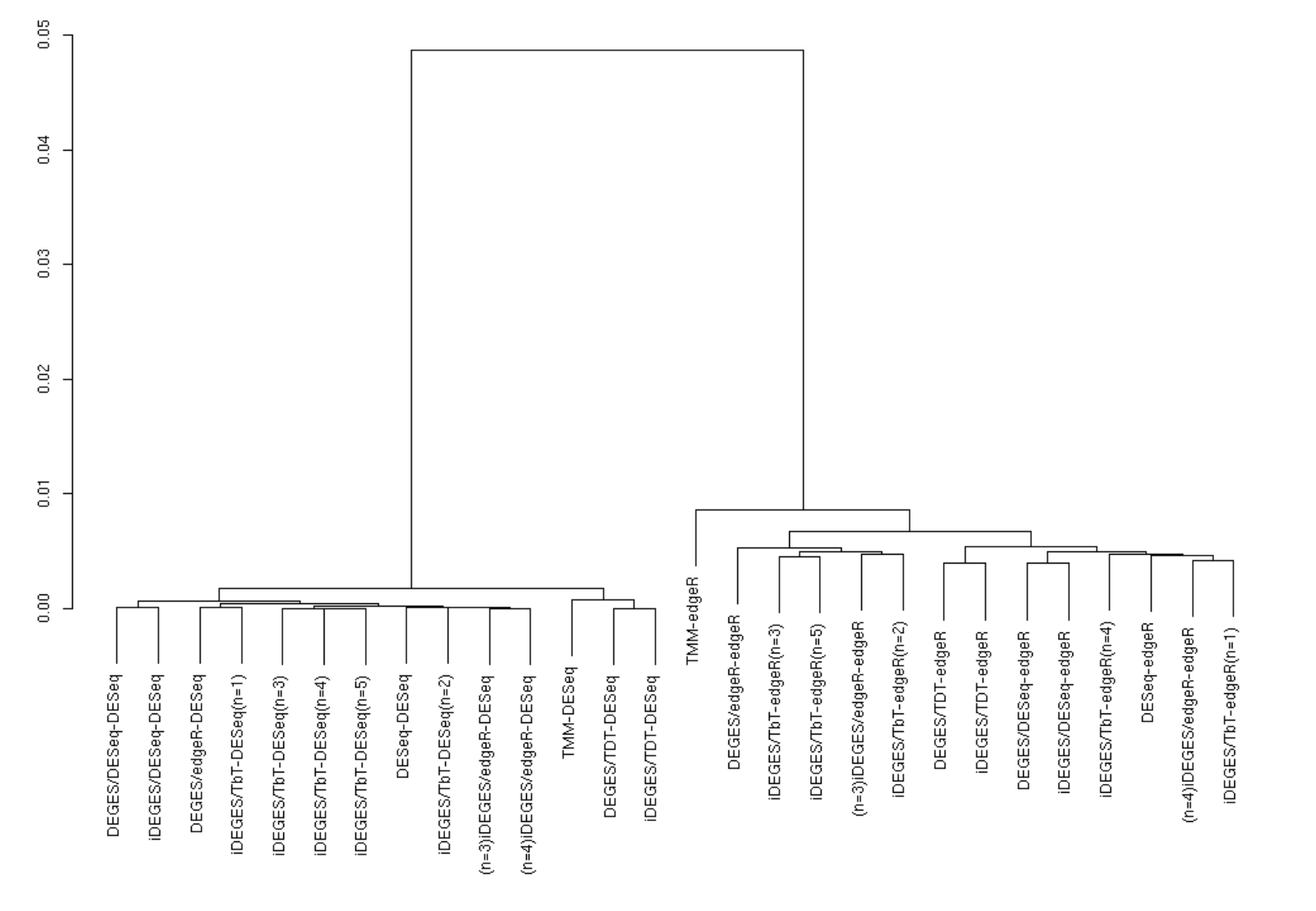

Supplement: Additional file 6 — Dendrogram of average-linkage hierarchical clustering for a katz.mouse dataset. Legends are basically the same as those in Figure 3. Gene lists having iteration numbers on the right side correspond to the results of the iDEGES/TbT-YYY combinations: the iDEGES/TbT pipeline for this dataset converged after the fifth iteration (i.e., NC = 5; see “Sheet 2” in Additional file 5). It can be seen that the ranked gene lists obtained from the same combination with different iteration numbers (i.e., the five lists obtained from the iDEGES/TbT-YYY combination with n = 1 ~ 5) are quite similar to the lists obtained from the other XXX-YYY combinations if YYY is the same. [file 1471-2105-14-219-S6.ppt]
